# Coverage statistics for sequence census methods

**DOI:** 10.1186/1471-2105-11-430

**Published:** 2010-08-18

**Authors:** Steven N Evans, Valerie Hower, Lior Pachter

**Affiliations:** 1 Department of Mathematics, University of California, Berkeley, California, USA; 2 Department of Statistics, University of California, Berkeley, California, USA; 3 Department of Molecular and Cell Biology, University of California, Berkeley, California, USA

## Abstract

**Background:**

We study the statistical properties of fragment coverage in genome sequencing experiments. In an extension of the classic Lander-Waterman model, we consider the effect of the length distribution of fragments. We also introduce a coding of the *shape *of the coverage depth function as a tree and explain how this can be used to detect regions with anomalous coverage. This modeling perspective is especially germane to current high-throughput sequencing experiments, where both sample preparation protocols and sequencing technology particulars can affect fragment length distributions.

**Results:**

Under the mild assumptions that fragment start sites are Poisson distributed and successive fragment lengths are independent and identically distributed, we observe that, regardless of fragment length distribution, the fragments produced in a sequencing experiment can be viewed as resulting from a two-dimensional spatial Poisson process. We then study the successive jumps of the coverage function, and show that they can be encoded as a random tree that is approximately a Galton-Watson tree with generation-dependent geometric offspring distributions whose parameters can be computed.

**Conclusions:**

We extend standard analyses of shotgun sequencing that focus on coverage statistics at individual sites, and provide a null model for detecting deviations from random coverage in high-throughput sequence census based experiments. Our approach leads to explicit determinations of the null distributions of certain test statistics, while for others it greatly simplifies the approximation of their null distributions by simulation. Our focus on fragments also leads to a new approach to visualizing sequencing data that is of independent interest.

## Background

The classic "Lander-Waterman model" [1] provides statistical estimates for the read depth in a whole genome shotgun (WGS) sequencing experiment via the Poisson approximation to the Binomial distribution. Although originally intended for estimating the redundancy when mapping by fingerprinting random clones, the Lander-Waterman model has served as an essential tool for estimating sequencing requirements for modern WGS experiments [2]. Further-more, although it makes a number of simplifying assumptions (e.g. fixed fragment length and uniform fragment selection) that are violated in actual experiments, extensions and generalizations [3-9] have continued to be developed and applied in a variety of settings.

The advent of "high-throughput sequencing", which refers to massively parallel sequencing technologies has greatly increased the scope and applicability of sequencing experiments. With the increasing scope of experiments, new statistical questions about coverage statistics have emerged. In particular, in the context of *sequence census methods*, it has become important to understand the *shape *of coverage functions.

Sequence census methods [10] are experiments designed to assess the content of a mixture of molecules via the creation of DNA fragments whose abundances can be used to infer those of the original molecules. The DNA fragments are identified by sequencing, and the desired abundances inferred by solution of an inverse problem. An example of a sequence census method is ChIP-Seq. In this experiment, the goal is to determine the locations in the genome where a specific protein binds. An anti-body to the protein is used to "pull down" fragments of DNA that are bound via a process called chromatin immunoprecipitation (abbreviated by ChIP). These fragments form the "mixture of molecules" and after purifying the DNA, the fragments are determined by sequencing. The resulting sequences are compared to the genome, leading to a *coverage function *that records, at each site, the number of sequenced fragments that contained it. As with many sequence census methods, "noise" in the experiment leads to random sequenced fragments that may not correspond to bound DNA, and therefore it is necessary to identify regions of the coverage function that deviate from what is expected in the "null" situation when only noise is present. Finding peaks that are extreme requires a definition of "extreme" in the sense of some test statistic taking a large value as well as a probability model for the coverage process that leads to the null distribution of the test statistic and hence to means for calibrating what values of the test statistic are improbably large in the null regime. The height of a peak is one obvious statistics, but we hope to get more discriminating procedures by also considering a suitably defined numerical summary of the shape of a peak. Indeed, the shape-based methods presented here have been used to develop a peak-caller--T-PIC--for the ChIP-Seq assay [11].

The purpose of this paper, however, is not to develop methods for data analysis, but rather to present a null model for the shape of a coverage function that is of general utility. That is, we propose a definition for the shape of a coverage function in terms of the topology of a tree. We describe a random instance assuming that fragments are selected at random from a genome, with lengths of fragments given by a known distribution. We indicate how our description can be used to either compute analytically or approximate via simple Monte Carlo simulation the distributions of quantities of interest in a data analysis.

## Methods

In this section, we use some specialized mathematical terminology and notation that the reader may be unfamiliar with. We feel it is important to include this in order to make our statements rigorous and mathematically correct. We will give the definitions of some of the concepts and a general idea of others, but first we set some notation. The symbols ℝ,ℤ, and ℤ_≥0 _stand for the real numbers, integers, and non-negative integers (respectively), and the elements of a set can be listed inside curly braces, for instance *A *= {1,2,3}.

### The shape of a fragment coverage function

We begin by explaining what we mean by a *coverage function*. Given a genome of length *N*, a coverage function is a function *f *: {1, ..., *N*} → ℤ_≥0_. The interpretation of this function is that *f*(*i*) is the number of sequenced fragments obtained from a sequencing experiment that cover position *i *in the genome. Because *N *is very large, we work with the set ℝ and redefine a coverage function as *f *: ℝ → ℤ_≥0_, which simplifies our analysis. We next introduce an object that describes a sequence coverage function's shape. Our approach is motivated by recent applications of topology including persistent homology [12,13] and the use of critical points in shape analysis [14-16]. For a given coverage function *f *: ℝ → ℤ_≥0_, we will define a rooted tree, which is a particular type of directed graph with all the directed edges pointing away from the root. This tree *T*_*f *_is based on the *upper-excursion sets off *: *U*_*h*_: = {(*x,f*(*x*))|*f*(*x*) ≥ *h*},*h *∈ ℤ_≥0 _and keeps track of how the sets *U*_*h *_evolve as h decreases. Long paths in *T*_*f *_represent features of the coverage function that persist through many values of *h*.

Specifically, for each *h *∈ ℤ_≥0_, let *C*_*h *_denote the set of connected components of the upper-excursion set *U*_*h*_. That is, each element of *C*_*h *_is an interval *I *such that *f*(*x*) ≥ *h *for all *x *∈ *I *and if *J *is another interval for which *I *⊂ *J *and *J *≠ *I *(so that *J *strictly contains *I*), then *f*(*y*) <*h *for some *y *∈ *J*. We define the rooted tree *T*_*f *_= (*V*,*E*) as follows

• Vertices in *V *correspond to the connected components in the sets *C*_*h*_, with *h *ranging over all non-negative integers.

• (*i*, *j*) ∈ *E *provided their corresponding connected components $c_{i}\in C_{h_{i}}$ and $c_{j}\in C_{h_{j}}$ with *h*_*i *_<*h*_*j *_satisfy *h*_*i *_= *h*_*j*_-1 and *c*_*j *_⊂ *c*_*i*_.

**Figure 1:**
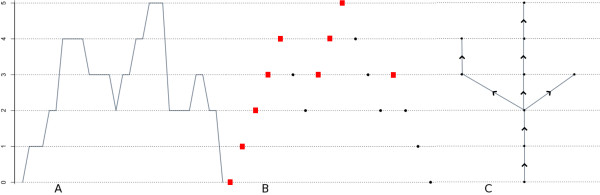
**A coverage function, lattice path excursion, and rooted tree**. A coverage function is depicted in (A) with its associated lattice path excursion (0,1,2,3,4,3,2,3,4,5,4,3,2,3,2,1,0) in (B). The lattice path excursion in (B) differs from the function (A) in that it records only the jumps of (A). It does not give any information regarding how long the function remains at each *y*-value. The rooted tree for the coverage function is in (C). The rooted tree is equivalent to the lattice path excursion (B). The red squares in (B) are the equivalence class representatives.

Note that the root of *T*_*f *_corresponds to the single connected component in *C*_0_. The tree *T*_*f *_is very similar to a contour tree [[14],§4.1], which is built using level sets of a function, and a join tree [17]. Indeed, suppose we ignore every vertex that is adjacent to only one vertex with greater height. Then, the remaining vertices of *T*_*f *_correspond to (equivalence classes of) local extrema of *f*. Each local maximum of *f *yields the birth of a new connected component as we sweep down through *h *∈ ℤ_≥0 _while a local minimum of *f *merges connected components. Since we do not require *f *to have distinct critical values (as is frequently assumed), the vertices in *T*_*f *_can have arbitrary (but assumed to be finite) degrees, as is depicted in Figure 1C.

In the sequel, we will use the following equivalent characterization that can be found in [[18], §2.3]. Given a coverage function *f *: ℝ → ℤ_≥0 _with *f*(*a*) = *f*(*b*) = 0 and *f*(*x*) > 0 for *x *∈ (*a*, *b*), we form an integer-valued sequence *x*_0_, ..., *x*_2*n *_that records the changes in height of *f *on the interval [*a,b*]. First, we note that while the coverage from one nucleotide to the next may jump by more than one, we can always extend the known function values to define a coverage function *f *on ℝ whose jumps are all one unit. In any case, for the probability model of the coverage function that we propose below, jumps of size greater than one occur with zero probability. Then, the sequence *x*_0_, ..., *x*_2*n *_consists of the *y *values that *f *travels through from *x*_0 _:= *f*(*a*) = 0 to *x*_2*n *_:= *f*(*b*) = 0 and satisfies

$$\begin{array}{l} x_{0}=x_{2n}=0,\\ x_{i}>0\;\;for\;\;0<i<2_{n},\\ \left|x_{i}-x_{i-1}\right|=1\;\;for\;\;1\leq i\leq 2n. \end{array}$$

Such a sequence is called a *lattice path excursion away from *0. Next, we define an equivalence relation on the set {0, 1, ..., 2*n*} by setting

$$i\equiv j\Leftrightarrow x_{i}=x_{j}=\underset{i\leq k\leq j}{\min}x_{k}.$$

The equivalence classes under this relation are in 1:1 correspondence with the connected components in the upper-excursion sets of *f*|_[*a*,*b*]_. One equivalence class is {0, 2*n*}, and if {*i*_1_, ..., *i*_*p*_} is an equivalence class with 0 <*i*_1 _<*i*_2 _< ... <*i*_*p *_then $x_{i_{1}-1}=x_{i_{1}}-1,$, whereas $x_{i_{q}-1}=x_{i_{q}}+1$ for 2 ≤ *q *≤ *p *Conversely, any index *i *with *x*_*i*-1 _= *x*_*i*_-1 is the minimal element of an equivalence class. We use the minimal element of each equivalence class as its representative. Thus, we can view the vertices of $T_{f{|}_{\left[a,b\right]}}$ as the set {0} ∪ {*i*|*x*_*i*-1 _= *x*_*i*_-1} Two indices *i*_1 _<*i*_2 _are adjacent in $T_{f{|}_{\left[a,b\right]}}$ provided $x_{i_{2}}=x_{i_{1}}+1$ and $x_{k}\geq x_{i_{1}}$ for *i*_1 _≤ *k *≤ *i*_2_. Figure 1 gives an example of a coverage function together with its lattice path excursion (0, 1, 2, 3, 4, 3, 2, 3, 4, 5, 4, 3, 2, 3, 2, 1, 0) and rooted tree. The minimal elements of each equivalence class in Figure 1B are depicted with red squares.

### Planar Poisson processes from sequencing experiments

In order to model random coverage along the genome thought of as a continuum, we adopt the perspective of the Lander-Waterman model and use a Poisson process to give random starting locations for the fragments. Specifically, we suppose that the left end-points of the fragments form stationary Poisson point process on ℝ with intensity *ρ*.

At each point of the Poisson point process we lay down an interval that has that point as its left end-point. The lengths of the successive intervals are independent and identically distributed with common distribution *μ*. We will use the notation *X *for a coverage function built from this process and *X*_*t *_for the height at a point *t*.

**Figure 2:**
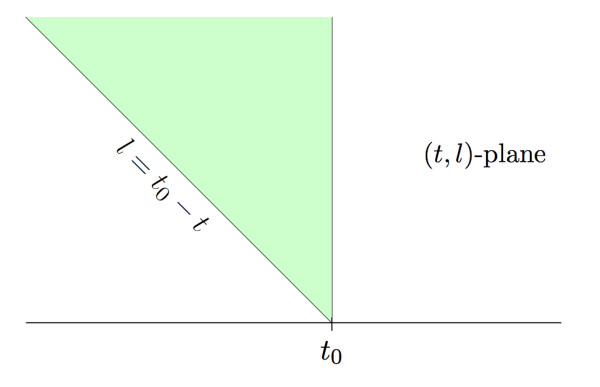
**A two dimensional view of a sequencing experiment**. A typical wedge in the (*t,l*) plane is shown. Each interval gives a point (*t*_*i*_,l_*i*_) in this plane where *t*_*i *_gives the start position of an interval and *l*_*i *_gives the length. The number of points in the green wedge gives the height $X_{t_{0}}$ of the coverage function at *t*_0_.

Let *t*_1_,*t*_2_, ... be the left-end points and *l*_1_,*l*_2_, ... be the corresponding lengths of intervals. The interval given by (*t*_*i*_, *l*_*i*_) will cover a nucleotide *t*_0 _provided *t*_*i *_≤ *t*_0 _and *t*_*i *_+ *l*_*i *_≥ *t*_0_. We can view this pictorially by plotting points {(*t*_*j*_,*l*_*j*_)} in the plane. Then $X_{t_{0}}$-- the number of intervals covering *t*_0_-- is the number of points in the wedge-shaped region in Figure 2.

Before defining a two-dimensional Poisson process, we note that the reader can think of *Borel *sets as being the "nice" subsets of ℝ^2 ^that *measures *are defined on, where a *measure *is a generalization of the area of a set. Any set the reader can imagine is almost certainly a Borel set and we include this terminology to maintain mathematical rigor - there are difficulties that arise in defining measures in a self-consistent manner on all subsets of ℝ^2 ^that don't arise if we restrict to Borel sets. We now recall the definition of a two-dimensional Poisson process and refer the reader to [[19],§6.13] or [[20], §2.4] for the details. Suppose is a locally finite measure on the Borel sets *ℬ *(ℝ^2^) (that is, Γ assigns finite mass to any bounded set). A random countable subset ∏ of ℝ^2 ^is called a *non-homogeneous Poisson process with mean measure *Γ if, for all Borel subsets *A*, the random variables *N*(*A*) := #(*A *⋂ ∏) satisfy:

1. *N(A) *has the Poisson distribution with parameter Γ(*A*), and

2. If *A*_1_, ..., *A*_*k *_are disjoint Borel subset of ℝ^2^, then *N*(*A*_1_), ..., *N*(*A*_*k*_) are independent random variables.

The following theorem is a theoretical statement about our null model for random fragment placement and is a consequence of [[21], Proposition 12.3]. The theorem and the work that follows from it will allow us to access the shape of random fragment placement by giving a description we can simulate.

**Theorem 1**. *The collection *{(*t*_*j*_,*l*_*j*_)} *of points obtained as described above is a non-homogeneous Poisson process with mean measure ρm *⊗ *μ.  Here m is Lebesgue measure (that is, length measure) on *ℝ.

The expected value of the coverage function $X_{t_{0}}$ at an arbitrary point *t*_0 _is the expected number of points that the Poisson process puts into the wedge-shaped region in Figure 2. By definition, this is the mass assigned to the wedge by the mean measure *ρm *⊗ *μ*That is, $E[X_{t_{0}}]$ = *ρm *⊗ *μ*Note that

$$\begin{array}{c} \rho m⊗\mu (\text{wedge})=\rho \int_{-\infty }^{t_{0}}\int_{t_{0}-u}^{\infty }\mu (dv)du\\ =\rho \int_{-\infty }^{t_{0}}\mu ((t_{0}-u,\infty ))du\\ =\rho \int_{0}^{\infty }\mu ((s,\infty ))ds\\ =\rho \int_{0}^{\infty }s\mu (ds), \end{array}$$

where the last line follows from an integration-by-parts. Thus, $E[X_{t_{0}}]$] is the product of the intensity *ρ *and the mean length of a fragment.

**Remark: **The average height *E *[$X_{t_{0}}$] can be computed without the use of Theorem 1. We include the derivation above as a first illustration that properties of the coverage function can be understood in terms of the two-dimensional Poisson process.

### Fragment lengths have a general distribution

To use the shape of fragment coverage in a data analysis, one needs to understand the distribution of the shape when fragments are laid down according to the null model described above. In particular, one is interested in the probability of seeing shapes associated with trees that have a height exceeding some high level. One way of doing this would be to first simulate a very long stretch of the two-dimensional Poisson process, determine the coverage function, construct the trees for peaks that exceed a high level, compute our shape statistic for each tree, and then record the empirical distribution of the resulting values. However, peaks that exceed high levels occur very infrequently and so we would need to simulate infeasibly long stretches of the Poisson process in order to determine the probabilities we are interested in with reasonable accuracy. Thus, in this section we propose a Markov approximation that lets us start at high levels (rather than wait for them to appear in simulations of the Poisson process). The corresponding trees are distributed as Galton-Watson trees with generation-dependent geometric offspring distributions and these are easy to simulate. In the Results and Discussion section, we compare this approximation to that obtained by simulating the Poisson process for fixed length fragments.

Suppose that we have a general distribution *μ*for the fragment lengths. The discrete-time stochastic process that records the values of *X *at its successive jumps is typically not a Markov chain (although, as we illustrate in the Results and Discussion section, it is if the distribution *μ*is exponential), but we will compute the conditional probability that *X *takes the values *k *± 1 at its next jump given that it currently has the value *k *and use the discrete-time Markov chain with transition probabilities given by these conditional probabilities as an approximation for the actual process of successive values of *X*. More precisely, we observe *X *at some fixed "time" -which might as well be 0 because of stationarity, and ask for the conditional probabilities given *X*_0 _that the next jump of *X *will be upwards to *X*_0 _+ 1 or down-wards to *X*_0_-1. Let *T *denote the time until the next fragment comes along. This random variable has an exponential distribution with rate *ρ *and is independent of *X*_0 _[[20], §2.1]. If we condition on *X*_0 _= *k*, the two-dimensional Poisson point process must have *k *points in the region

**Figure 3:**
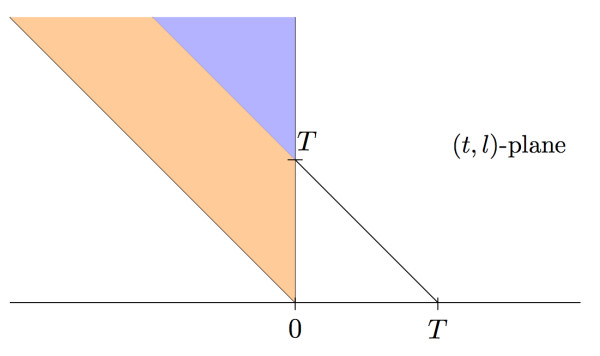
**A wedge from the planar Poisson process**. The intervals that correspond to points in both the blue and orange regions contribute to the height *X*_0_. Any point in the orange region would "die" before *T *while points in the blue region contribute to the height *X*_*T*_.

A:={(t,l):−∞<t≤0,−t<l<∞},

depicted in Figure 3. Conditionally, these *k *points in *A *have the same distribution as *k *points chosen at random in *A *according to the probability measure

$$\begin{array}{lll} \frac{\rho m⊗\mu (B)}{\rho m⊗\mu (A)} & \text{for} & B\subset A \end{array}.$$

However, in order that the next jump after 0 is up-wards, the two-dimensional Poisson point process must have no points in the orange region

{(t,l):−∞<t≤0,−t<l<T−t}

in Figure 3 as these fragments end before time *T*. This leaves the *k *points lying in the blue region

{(t,l):−∞<t≤0,T−t≤l<∞},

which occurs with probability ${\left(\frac{\rho \int_{T}^{\infty }\mu ((u,\infty ))du}{\rho \int_{0}^{\infty }\mu ((u,\infty ))du}\right)}^{k}$.

Thus, conditional on *X*_0 _= *k*, the probability that the next jump will be upwards is

$$\int_{0}^{\infty }{\left(\frac{\int_{t}^{\infty }\mu ((u,\infty ))du}{\int_{0}^{\infty }\mu ((u,\infty ))du}\right)}^{k}\rho e^{-\rho t}dt.$$

Write *p*(*k*) for this quantity. To build trees, we are interested in the jumps of the coverage function, and hence we define a discrete-time Markov chain on the nonnegative integers with transition probabilities

$$P(i,j)=\{\begin{array}{ll} 1, & \text{if}\;\text{i}=0\text{ and }j=1,\\ p(i), & \text{if}\;i\geq 1\text{ and }j=i+1,\\ 1-p(i), & \text{if}\;i\geq 1\text{ and }j=i-1,\\ 0, & \text{otherwise}. \end{array}$$

Suppose now we have a lattice path excursion starting at 0. Given a vertex *v *of the associated tree at height *k*, we are interested in the number of offspring (at height *k *+ 1) of this vertex. Suppose *i*_0 _is the minimal equivalence class representative for vertex *v*, and suppose *i*_0 _the equivalence class of *i*_0 _is {*i*_0_, *i*_1_,..., *i*_*n*_} with *i*_0 _<*i*_1 _< ... <*i*_*n*_. Then, we have $x_{i_{r}}=k$ for 0 ≤ *r *≤ *n*, $x_{i_{r}+1}=k+1$ for 0 ≤ *r *≤ *n *- 1, $\;x_{i_{n}+1}=k-1$, and *x*_*t *_>*k *for *i*_0 _<*t *<*i*_*n *_with *t *≠ some *i*_*r*_. From the Markov property, for 0 ≤ *j *≤ *n *we have the equations

$$\begin{array}{l} \mathbb{P}\{x_{i_{j}+1}=k+1|x_{i_{j}}=k\}=p(k)\text{ and}\\ \mathbb{P}\{x_{i_{j}+1}=k-1|x_{i_{j}}=k\}=1-p(k). \end{array}$$

The resulting tree is a Galton-Watson tree with generation-dependent offspring distributions (see [22-25] for more on Galton-Watson trees). Indeed, the probability a vertex at height *k *has *n *offspring is given by

(1) $$p{\left(k\right)}^{n}(1-p(k)),$$

which is the probability of *n *failures before the first success in a sequence of independent Bernoulli trials where the probability of success equals 1-*p*(*k*). The utility of Equation 1 is that it allows one to (approximately) simulate trees for peaks that exceed a high level under the null model, making it possible to compare trees built from actual data to those formed by random fragment placement.

We close this section by processing another feature of the trees (under the null model) that we can compute using our Markov approximation. Let *r*(*i*, *j*) be probability that our Markov chain started in height *i *reaches height *j *before it hits height 0. We have the relations

(2) r(i, j)=p(i)r(i+1, j)+(1-p(i))r(i-1, j)

with the boundary conditions *r*(*i*, *j*) = 1 and *r*(0, *j*) = 0: Next, given a height *H*, let $Y_{n}:=\frac{r(n,H)}{r(1,H)}$, for 1 ≤ *n *≤ *H*. Using equation (2), we have

$$Y_{n+1}=\frac{Y_{n}+(p(n)-1)Y_{n-1}}{p(n)}$$

for 2 ≤ *n *≤ *H *- 1 with *Y*_1 _= 1, $Y_{2}=\frac{1}{p(1)}$. We may solve inductively for *Y*_*H *_and obtain $r(1,H)=\frac{1}{Y_{H}}$. The quantity *r*(1,*H*) gives the probability that a tree corresponding to a single lattice path excursion away from 0 and coming from the null model is at least as tall as height *H*. Note that this type of tree comes from a block where the coverage function rises from 0 and then back again-often referred to as an island or contig. This probability can be used to do an initial "filtering" of peaks in a data analysis: one first concentrates on peaks that exceed some height that is calibrated using a knowledge of *r*(1,*H*) and then computes the shape statistic and associated *p*-values for just those peaks. As an example, Figure 5 in the Results and Discussion section shows *r*(1,*H*) plotted for the fixed fragment length.

## Results and Discussion

### Fragment lengths have the exponential distribution

When the distribution *μ*of fragment lengths is exponential with rate λ, our Markov approximation is exact, as shown below. In this case, we have *μ*((*s*,∞)) = ℙ{*l *>*s*} = *e*^-λ *s*^and

$$E(X_{t})=\rho \int_{0}^{\infty }e^{-\lambda s}ds=\frac{\rho }{\lambda }.$$

**Claim 1**. *The process X is a stationary, time-homogeneous Markov process*.

Proof. It is clear that *X *is stationary because of the manner in which it is constructed from a Poisson process on ℝ^2 ^that has a distribution which is in-variant under translations in the *t *direction; that is, the random set {(*t*_*i*_,*l*_*i*_)} has the same distribution as {(*t*_*i *_+ *t,l*_*i*_)} for any fixed *t *∈ ℝ. Since *μ*is exponential, it is memoryless, meaning for any interval length *l *with an exponential distribution

ℙ{l>a+b|l>a}=ℙ{l>b}.

This means that probability that an interval covers *t*_2 _knowing that it covers *t*_1 _is the same as the probability that an interval starting at *t*_1 _covers *t*_2_. Thus, the probability that $X_{t_{2}}=k$ given *X*_*t *_for at *t ≤ t*_1 _only depends on the value of $X_{t_{1}}$. Indeed, in terms of time, $\mathbb{P}\{X_{t_{2}}=k|X_{t_{1}}=k^{\prime }\}$ depends only on *t*_2 _-*t*_1._

More specifically, X is a birth-and-death process with birth rate *β*(*k*) = *ρ *in all states *k *and death rate *δ*(*k*) = *k*λ in state *k *≥ 1. The jumps of *X *are given by a discrete-time Markov chain with transition matrix

$$P(i,j)=\{\begin{array}{ll} 1, & \text{if}\;i=0\text{ and }j=1,\\ \frac{\rho }{\rho +i\lambda }, & \text{if}\;i\geq 1\text{ and }j=i+1,\\ \frac{i\lambda }{\rho +i\lambda }, & \text{if}\;i\geq 1\text{ and }j=i-1,\\ 0, & \text{otherwise}, \end{array}$$

and we have the probability a vertex at height *k *has *n *offspring is

$${\left(\frac{\rho }{\rho +\lambda k}\right)}^{n}\frac{\lambda k}{\rho +\lambda k}.$$

Note that as the exponential distribution is the only distribution with the memoryless property, we lose the Markov property when μis not exponential.

### Fragments have a fixed length

Suppose *μ*is the point mass at *L *(that is, all fragment lengths are *L*). Then

$$\mu ((u,\infty ))=\{\begin{array}{cc} 1, & u<L\\ 0, & u\geq L \end{array},$$

and

$$\int_{t}^{\infty }\mu ((u,\infty ))du=\{\begin{array}{ll} \int_{t}^{L}du=L-t, & t<L\\ 0, & t\geq L. \end{array}$$

This gives

$$\begin{array}{c} p(k)\;=\int_{0}^{L}\frac{{\left(L-t\right)}^{k}}{L^{k}}\rho e^{-\rho t}dt\\ =\int_{0}^{1}w^{k}\rho e^{-\rho (L-Lw)}Ldw\\ =\theta e^{-\theta }\int_{0}^{1}w^{k}e^{\theta w}dw \end{array}$$

for *k *≥ 1, where *θ*: = *ρL *= $E[X_{0}]$. We integrate by parts and find that *p*(*k*) = *θe*^*-θ*^*q*(*k*) where

$$\begin{array}{c} q(k)={\frac{w^{k}e^{\theta w}}{\theta }|}_{w=0}^{w=1}-\frac{k}{\theta }\int_{0}^{1}w^{k-1}e^{\theta w}dw\\ =\frac{e^{\theta }}{\theta }-\frac{k}{\theta }q(k-1) \end{array}$$

for *k *≥ 2, which yields the recursion

(3) $$p(k)=1-\frac{k}{\theta }p(k-1),$$

for *k *≥ 2 with $p(1)=1-\frac{1}{\theta }+\frac{e^{-\theta }}{\theta }$. solving explicitly, we obtain

(4) $$p(k)=k!\left(\sum_{j=0}^{k}\frac{{\left(-1\right)}^{k-j}}{j!\theta^{k-j}}+\frac{{\left(-1\right)}^{k-1}e^{-\theta }}{\theta^{k}}\right)$$

for *k *≥ 1. Below we verify that Equation (4) satisfies the recursion in Equation (3):

$$\begin{array}{l} 1-\frac{k}{\theta }p(k-1)\\ =1-\frac{k!}{\theta }\left(\sum_{j=0}^{k-1}\frac{{\left(-1\right)}^{k-1-j}}{j!\theta^{k-1-j}}+\frac{{\left(-1\right)}^{k-2}e^{-\theta }}{\theta^{k-1}}\right)\\ =k!\left(\frac{1}{k!}+\sum_{j=0}^{k-1}\frac{{\left(-1\right)}^{k-j}}{j!\theta^{k-j}}+\frac{{\left(-1\right)}^{k-1}e^{-\theta }}{\theta^{k}}\right)\\ =k!\left(\sum_{j=0}^{k}\frac{{\left(-1\right)}^{k-j}}{j!\theta^{k-j}}+\frac{{\left(-1\right)}^{k-1}e^{-\theta }}{\theta^{k}}\right)\\ =p(k). \end{array}$$

**Figure 4:**
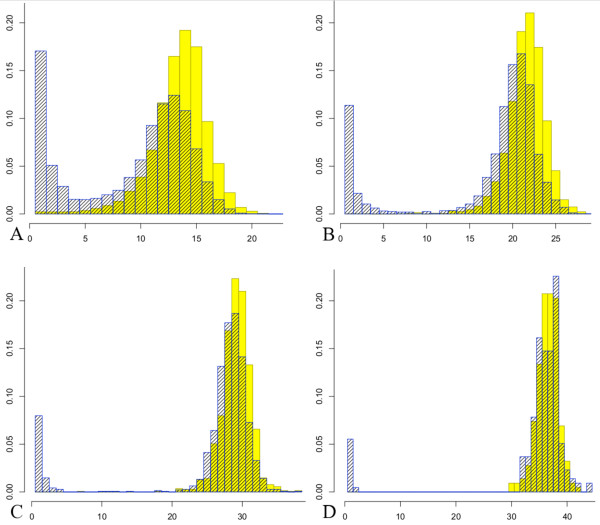
**Comparison of the Poisson process and Markov approximation in terms of tree height**. Histograms of the densities for tree height are shown for trees built from a simulated Poisson process (solid yellow) and Galton-Watson trees from the Markov approximation (blue striped) for the case of fixed fragment lengths. Each tree corresponds to one lattice path excursion away from 0 (also referred to as sequence islands or contigs). The simulations include average height *θ *= 6 with 14,466 trees simulated for each type (A), *θ *= 9 with 3,551 trees simulated for each type (B), *θ *= 12 with 1,429 trees simulated for each type (C), and *θ *= 15 with 217 trees simulated for each type (D).

**Figure 5:**
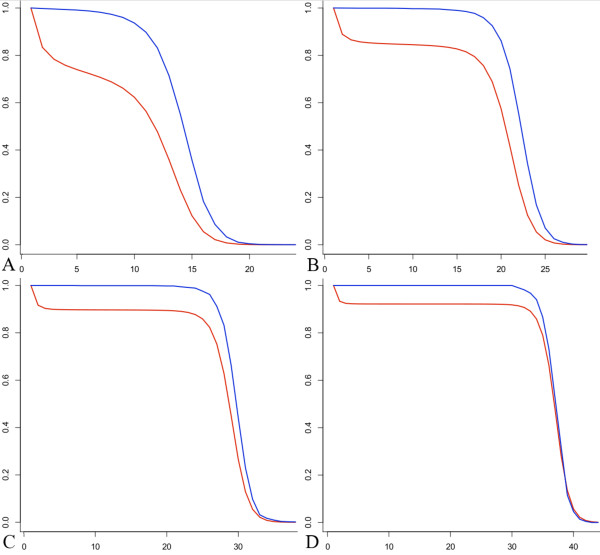
**Comparison of trees built from the Poisson process with the probability *r*(1, H)**. The function *r*(1,*H*) = Π{Galton-Watson tree has height ≥ *H*} is plotted in red. Using trees from a simulated Poisson process, the function Π{tree from simulated Poisson process has height ≥ *H*} is plotted in blue. The plots include average height *θ *= 6 (A), *θ *= 9 (B), *θ *= 12 (C) and *θ *= 15 (D) for the case of fixed fragment lengths.

Next, we compare the trees built from the Markov approximation to the trees arising from the Poisson process when fragments have a fixed length. We simulate trees with average height *θ *= 6,9,12, and 15 using both the Poisson process and the Markov approximation. The histograms in Figure 4 show the densities of simulated trees for the Markov approximation (blue striped bars) and for the Poisson process (yellow solid bars) for *θ *= 6,9,12, and 15. Additionally, the plots in Figure 5 depict the probabilities *r*(1,*H*) (in red) and Π{tree from simulated Poisson process has height ≥ *H*} (in blue). These figures illustrate that, for large *θ*, the Markov approach seems like a reasonable approximation.

**Figure 6:**
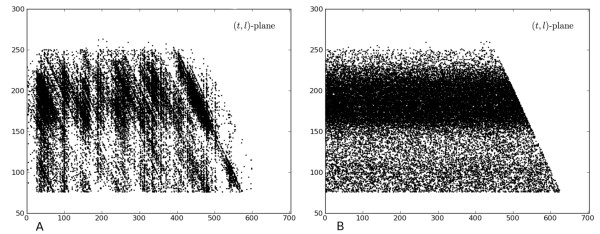
**Examples of sequencing in the (*t,l*) plane**. (A) Fragments from a sequencing experiment shown in the (*t,l*) plane. (B) The spatial Poisson process resulting from fragments with the same length distribution as (A) but with position sampled uniformly at random.

Our observation that randomly sequenced fragments from a genome form a planar Poisson process in (*position, length*) coordinates has implications beyond the coverage function analysis performed in this paper. For example we have found that the visualization of sequencing data in this novel form is useful for quickly identifying instances of sequencing bias by eye, as it is easy to "see" deviations from the Poisson process. An example is shown in Figure 6 where fragments from an Illumina sequencing experiment are compared with an idealized simulation (where the fragments are placed uniformly at random). Specifically, paired-end reads from an RNA-Seq experiment conducted on a GAII sequencer were mapped back to the genome and fragments inferred from the read end locations. Bias in the sequencing is immediately visible, likely due to non-uniform PCR amplification [26] and other effects.

The "shape" we have proposed for coverage functions was motivated by persistence ideas from topological data analysis (TDA). In the context of TDA, our setting is very simple (1-dimensional), however unlike what is typically done in TDA, we have provided a detailed probabilistic analysis that can be used to construct a null hypothesis for coverage-based test statistics. For example, computing test statistics [27] based on the trees constructed from coverage functions and comparing those to the statistics expected from the Galton-Watson trees has been used to determine protein binding sites in ChIP-Seq assay [11]. It should be interesting to perform similar analyses with high-dimensional generalizations for which we believe many of our ideas can be translated. There are also biological applications, for example in the analysis of Chip-Seq experiments [11], as previously mentioned.

## Conclusions

We believe that the study of sequence coverage functions that we have initiated may be of use in the analysis of many sequence census methods. The number of proposed protocols used in such methods has exploded in the past two years, as a result of dramatic drops in the price of sequencing. For example, in January 2010, the company Illumina announced a new sequencer, the HiSeq 2000, that they claim "changes the trajectory of sequencing" and can be used to sequence 25 Gb per day. Al-though technologies such as the HiSeq 2000 were motivated by human genome sequencing a surprising development has been the fact that the majority of sequencing is in fact being used for sequence census experiments [10]. The vast amounts of sequence being produced in the context of complex sequencing protocols, means that a detailed probabilistic under-standing of random sequencing is likely to become increasingly important in the coming years.

## Authors' contributions

LP proposed the problem of understanding the random behaviour of coverage functions in the context of sequence census methods. VH investigated the coverage function and lattice path excursions based on ideas from topological data analysis. SE developed the probability theory and identified the relevance of Theorem 1. SNE, VH and LP worked together on all aspects of the paper and wrote the manuscript. All authors read and approved the final manuscript.
